# Fracture resistance of endodontically treated teeth restored with Zirconia filler containing composite core material and fiber posts

**DOI:** 10.12669/pjms.326.11282

**Published:** 2016

**Authors:** Zaid Al Jeaidi

**Affiliations:** Dr. Zaid Al Jeaidi, Assistant Professor, Conservative Dental Science Department, College of Dentistry, Prince Sattam Bin Abdulaziz University, Al-Kharj, Saudi Arabia

**Keywords:** Bulk fill composite, Endodontic teeth, Restoration, Fracture resistance, Zirconia filler

## Abstract

**Objectives:**

To assess the fracture resistance of endodontically treated teeth with a novel Zirconia (Zr) nano-particle filler containing bulk fill resin composite.

**Methods:**

Forty-five freshly extracted maxillary central incisors were endodontically treated using conventional step back preparation and warm lateral condensation filling. Post space preparation was performed using drills compatible for fiber posts (Rely X Fiber Post) on all teeth (n=45), and posts were cemented using self etch resin cement (Rely X Unicem). Samples were equally divided into three groups (n=15) based on the type of core materials, ZirconCore (ZC) MulticCore Flow (MC) and Luxacore Dual (LC). All specimens were mounted in acrylic resin and loads were applied (Universal testing machine) at 130° to the long axis of teeth, at a crosshead speed of 0.5 mm/min until failure. The loads and the site at which the failures occurred were recorded. Data obtained was tabulated and analyzed using a statistical program. The means and standard deviations were compared using ANOVA and Multiple comparisons test.

**Results:**

The lowest and highest failure loads were shown by groups LC (18.741±3.02) and MC (25.16±3.30) respectively. Group LC (18.741±3.02) showed significantly lower failure loads compared to groups ZC (23.02±4.21) and MC (25.16±3.30) (p<0.01). However groups ZC (23.02±4.21) and MC (25.16±3.30) showed comparable failure loads (p=0.23).

**Conclusions:**

Fracture resistance of endodontically treated teeth restored with Zr filler containing bulk fill composite cores was comparable to teeth restored with conventional Zr free bulk fill composites. Zr filled bulk fill composites are recommended for restoration of endodontically treated teeth as they show comparable fracture resistance to conventional composite materials with less catastrophic failures.

## INTRODUCTION

Endodontic treatment of teeth with pulpal involvement results in considerable loss of tooth structure, and tooth weakness.^1^ In addition reduced availability of remaining tooth structure makes the task of restoration further challenging. Root posts are used to provide retention for the core materials, which are predictably delivered with contemporary resin composites.^2^,^3^ Therefore, the mechanical and physical properties of the buildup core material critically affects the successful clinical performance of restored endodontic teeth.^4^

Nano particle size fillers have been introduced in composites to provide improved physical and mechanical properties.^5^-^7^ (10,11,12) Nano fillers frequently contain Zr particles, known to enhance the biocompatibility, durability and mechanical properties of resin composites.^8^,^9^ Zr is an exceptionally stable and durable material, with excellent biocompatibility and mechanical properties.^9^ Zr fillers in bulk fill resin composites have been recently introduced in the form of ZirconCore (Harvard Dental International, GmbH, Hoppegarten, Germany) for core build up purpose in endodontically treated teeth. It is observed through laboratory based studies, that Zr nano particle containing restorative composite resins have shown better compressive strengths in comparison to silica and barium based micro and macro-filled composites.^6^

Recently, it was reported that Zr nano-particle filler containing bulk fill composite build up materials show higher compressive strength values as compared to conventional bulk fill materials.^10^ Therefore, it is hypothesized that the incorporation of Zr nano particle filler in a bulk fill composite build-up material could improve the fracture resistance of bulk fill resin composite build up materials. To our knowledge from indexed literature, studies assessing the impact of Zr filler containing bulk fill material on the fracture resistance of endodontically treated teeth is limited. Therefore the aim of the study was to assess the fracture resistance of endodontically treated teeth with novel Zr nano-particle filler containing bulk fill resin composite.

## METHODS

Forty-five freshly extracted maxillary central incisors (of similar dimensions) without any physical deficiency were selected for the study and stored in normal saline. All samples (45 teeth) were divided into 3 groups (n=15), depending on the types of core build-up material [ZirconCore (ZC), MulticCore Flow (MC) and Luxacore Dual (LC)] after root canal treatments and restoration with fiber posts.

For endodontic treatment, all teeth were sectioned 2-mm coronal to the cemento-enamel junction (for 2mm ferrule) and were prepared with a bur to produce a 1.5mm deep chamfer finish margin. Using a round diamond bur, pulp chamber was accessed, pulp was extirpated, patency was achieved (15K file) and irrigation with 5.25% sodium hypochlorite was performed. The working length was kept 1mm short of apex and the canals were prepared (file # 15 to #60) using conventional technique. All root canals were irrigated and dried prior to obturation using a non-eugenol sealer (SealApex, KavoKerr Group, Orange, CA 92867, United States) and compatible gutta percha (GP) cones with warm lateral condensation method. Post space was prepared using universal drills compatible for fiber posts (Rely X Fiber Post, 3M, ESPE, St. Paul, MN, USA) with color code red (diameter: apical 1.60mm, coronal 0.80mm, taper: 8%). Using a silicone stopper on drills, post space was prepared to 10mm depth from coronal dentin. The compatible drill sequence was employed for red post size (diameter: apical 1.60mm, coronal 0.80mm, taper: 8%). The posts were cut to appropriate lengths to produce an assembly of posts being 8mm in the root canal, 2mm of ferrule and 3mm above the prepared coronal dentin. A self etch adhesive cement (Rely X Unicem, Self etch cement, 3M, ESPE, St. Paul, MN, USA) was used for post cementation in all groups. The post space was irrigated with sodium hypochlorite 5.25% (5 ml syringe), followed with water rinse and drying with paper points. The fiber posts were cleaned with alcohol and cemented using self etch adhesive resin cement (Rely X Unicem, Self etch cement, 3M, ESPE, St. Paul, MN, USA). Cement was applied to the apical half of the post in a wiping motion and placed under a standard load of 1 Kilograms. The posts were light cured for 40 seconds (LED-650 mWcm-^2^) and allowed to self-cure for five minutes. For list of materials see appendix A.

All specimen teeth were divided into three groups (n=15) and core build ups were performed using ZirconCore (ZC) (Harvard Dental International, GmbH, Hoppegarten, Germany), MulticCore Flow (MC) (Ivoclar Vivadent Schaan Liechtenstein) and Luxacore Dual (LC) (DMG America, Englewood, New Jersey). The groups were designated as ZC, MC and LC. Core build-ups were performed using a core former and the materials were applied using the recommended bonding agents and protocols. A metal mould was utilized to mount restored teeth in auto-polymerizing acrylic resin (keeping margin at 3mm above the resin). With the use of universal testing machine with mounting jig, controlled loads were applied to the specimen samples at 130° to the long axis, at a crosshead speed of 0.5 mm/min until failure. The loads and the site at which the failures occurred were recorded. All failure sites were assessed for either failure above the CEJ (with some coronal dentin) (A) or below the CEJ with no coronal tooth structure remaining (B). All data was tabulated in an excel sheet and analyzed using SPSS (Statistical Program for Social Sciences). The means and standard deviations were compared using ANOVA.

## RESULTS

All data passed the normality test using the Kolmogorov and Smirnov (KS) test. The assessed mean root lengths and width at CEJ were 15.83±1.68 and 6.47±0.59 respectively. The lowest and highest failure loads (N) were shown by groups LC (18.741±3.02) N and MC (25.16±3.30) respectively. The means and standard deviations of failure loads achieved in each of the experimental groups are summarized in Table-I. Analysis of variance showed statistically significant difference among the study groups (p<0.001) (Table-I). Using Tukey post hoc test statistical comparison between different groups was completed (Table-II). Group LC (18.741±3.02) showed significantly lower failure loads compared to groups ZC (23.02±4.21) and MC (25.16±3.30) (p<0.01). However groups ZC (23.02±4.21) and MC (25.16±3.30) showed comparable failure loads (p=0.23).

**Table-I:** Failure loads among study groups (ANOVA).

Experimental Groups	Means	SD	P value
ZC	23.020	4.214	<0.001*
MC	25.163	3.305	
LC	18.741	3.028	

ZC zircon core, MC Multicore, LC Luxacore,

* Highly significant, SD standard deviations.

**Table-II:** Comparison of means using Tukey Post Hoc Test at 95% CI.

Experimental Groups	Means	P value
ZC vs MC	23.020 vs 25.163	0.235ns
ZC vs LC	23.020 vs 18.741	<0.01*
MC vs LC	25.163 vs 18.741	< 0.001**

ZC zircon core, MC Multicore, LC Luxacore, ns, not significant.

* significant,

** highly significant.

When comparing specimen failure types among different groups (Table-III), all failures in ZC group were type A (failure above the CEJ, with some coronal dentin). However in groups LC and MC, 86.6% and 66.6% of type A failures were recorded respectively.

**Table-III:** Type of failures among the experimental groups.

Experimental Groups	Type of specimen failures- No (%)	

	A	B
ZC	15 (100)	0 (0.00)
MC	10 (66.6)	5 (33.3)
LC	13 (86.66)	2 (13.33)

ZC zircon core, MC Multicore, LC Luxacore, % Percentage. A, Failure above the CEJ, with some coronal dentin, B, Failure below the CEJ with no coronal tooth structure remaining.

## DISCUSSION

The success and longevity of endodontically treated teeth is related to an array of factors including, quality of endodontic treatment, remaining tooth structure and coronal restoration.^11^ The need for restoration types for endodontically treated teeth primarily depends on the quality and quantity of remaining dentin.^12^ In cases of extensive damage, the remaining tooth structure is replaced with a core restoration retained by a post to provide retention and resistance for the extra coronal restoration.^13^ Multiple, post and core systems are employed, however use of resin composite cores along with fiber posts are not only commonly used but in comparison also provide strength and esthetics.^14^ In the present study fracture resistance of endodontically treated teeth restored with fiber posts and multiple bulk fill resin composites was assessed.

All selected teeth were central incisors, with root lengths and widths being statistically comparable (p>0.01). A ferrule of 2mm and a chamfer margin of 1.5mm were prepared through all samples to simulate clinical conditions in anterior teeth with cervical failures, restored with extra-coronal full veneer crowns.^15^,^16^ The length and width of posts, along with there relative positioning in the roots were within the clinical recommendations.^17^ In addition, loads were applied at 130 degree on the palatal aspect of incisors to simulate natural occlusal contacts, at a crosshead speed of 0.05mm/min as recommended by previous studies.^18^,^19^

The present study was based on the hypothesis that endodontically treated teeth which are build up with Zr filler containing composite bulk fill material would show better fracture resistance compared to those restored with conventional bulk fill composites. However this hypothesis was unfounded, as specimens in ZC group showed comparable fracture resistance outcomes to those in MC group. Multiple reasons can be posed in this regard. For instance it is known that the quality of adhesive bond strength of composite resins to tooth structure has a critical impact on the overall strength of an endodontically treated tooth.^20^ In a recent study,^10^ it was proposed that zirconia particles fail to show a durable bond to silane coupling agent in the composite resin material. In addition, the authors reported significantly lower bond strength values for Zr filler containing bulk fill composites compared to conventional resin bulk fill materials.^10^

Interestingly, fracture resistance among ZC and MC groups were significantly greater than LC group specimens. A possible explanation for these outcomes could be associated with the type of bonding agents used in the present experiments. For optimum results the operators used the recommended bonding regime for the three different bulk fill materials (ZC, MC and LC) in the present study. In the ZC and MC groups total etch bonding regimes, with the use of Harvard bond TE Mono (Harvard Dental International, GmbH, Hoppegarten, Germany) and Adhese Universal (Ivoclar Vivadent, Schaan, Liechenstein) bonding agents were employed, respectively. However for the LC group specimen, the recommended self etching, Contax bonding agent (DMG, Chemisch-Pharmazeutische Fabrik GmbH, Hamburg, Germany) was applied. It is widely suggested that the bond strength, micro leakage, and bonding layer degradation varies with the use of self etch and total etch bonding agents.^21^-^23^ All these three properties could have potentially impacted the overall fracture resistance of endodontically treated teeth in LC group.

In the present study, failures were divided into salvageable (type A) and non salvageable (type B) failures.^24^,^25^ It was observed that specimen in MC group (with maximum failure loads) showed most (33.3%) type B failures. This may indicate that due to improved adhesive bond and mechanical strength of the materials (MC), stresses were transferred to the tooth, resulting in its fracture. However, all specimen in ZC group showed type A, salvageable failures. In the authors opinion this is related to the lower bond strength of the Zr filler containing material as shown previously.^10^ This also suggest in light of previous evidence that the stiffness of Zr filler containing composite and a relatively low adhesive bond would result in reversible failures of endodontically treated and restored teeth.

Findings of this study strongly suggest the clinical use of Zr filler bulk fill materials for buildup of endodontically treated teeth, as they showed comparable failure resistance to conventional non-Zr bulk fill materials (ZC vs MC, p>0.01). In addition, failures (salvageable) when occurred were above the CEJ, which allows for subsequent restoration of teeth.

## CONCLUSION

Within the limitations of the study, it can be concluded that fracture resistance of endodontically treated teeth restored with Zr filler containing bulk fill composite cores was comparable to teeth restored with conventional Zr free bulk fill composites. Zr filled bulk fill composites are recommended for restoration of endodontically treated teeth as they show comparable fracture resistance to conventional composite materials with less catastrophic failures.

**Appendix A:** (Composition of materials and equipment)

1. MultiCore Flow (Ivoclar Vivadent Schaan Liechtenstein)
***Composition:*** Microhybrid resin composite, the monomer matrix consists of dimethacrylate (29 wt %). The inorganic fillers are barium glass, ytterbiumtrifluoride, Ba-Al-fluorosilicate glass and highly dispersed silicon dioxide (70 wt %). Additional contents are catalysts, stabilizers and pigments (1 wt %).
2. LuxaCore Dual (DMG America, Englewood, New Jersey)
***Composition:*** Microhybrid resin composite, Barium glass 69%, pyrog. silica 3% in a Bis-GMA based matrix of dental resins.
3. ZirconCore (Harvard Dental International, GmbH, Hoppegarten, Germany)
***Composition:*** Nano hybrid composite resin, Dimethacrylates 35%, Starter 2%, Silica Filler 10%, Glass filler 55%, Zirconium dioxide 5%, Pigments 0,5%.
4. Rely X Unicem, Self etch cement, 3M, ESPE, St. Paul, MN, USA
5. SealApex, KavoKerr Group, Orange, CA 92867, United States
6. Fiber posts: Rely X Fiber Post, 3M, ESPE, St. Paul, MN, USA
7. Harvard bond TE Mono
***Composition:*** Modified acrylic acid, poly acrylic acid, methacrylates, catalysts, stabilizators in ethanol
8. Harvard Etch
***Composition:*** 37% phosphoric acid based on thixotropic gel
9. Adhese Universal, (Ivoclar Vivadent, Schaan, Liechenstein)
Acrylic acids, catalyst and stabilizers
10. Contax Bonding Agent (DMG, Chemisch-Pharmazeutische Fabrik GmbH, Hamburg, Germany) Self-etch. Polyacrylic acid, catalyst and stabilizers.
11. Universal testing machine (Model 4411; Instron Corp, Canton, Mass)
12. Light curing device (Bluephase ® C8, Ivoclar Vivadent, Schaan, Liechenstein)
13. TCS-30 (Thermocycling-testing-system, Certiga engineering solutions, Netherlands)
14. Digital caliper (Stainless Steel, Series-500, Mitutoyo, USA)
